# Anti-lipoapoptotic effect of *Artemisia capillaris* extract on free fatty acids-induced HepG2 cells

**DOI:** 10.1186/1472-6882-14-253

**Published:** 2014-07-19

**Authors:** Eungyeong Jang, Min-Hee Shin, Ki-Suk Kim, Yoomi Kim, Yun-Cheol Na, Hong-Jung Woo, Youngchul Kim, Jang-Hoon Lee, Hyeung-Jin Jang

**Affiliations:** 1 College of Korean Medicine, Institute of Korean Medicine, Kyung Hee University, Hoegi-dong, Dongdaemun-gu, Seoul 130-701, Republic of Korea; 2 Integrated Metabolomics Research Group, Seoul Center, Korea Basic Science Institute, 126-16 Anam-Dong, Seungbuk-Ku, Seoul 136-713, Republic of Korea

**Keywords:** *Artemisia capillaris* (AC), HepG2, Non-alcoholic steatohepatitis (NASH), Lipoapoptosis, c-Jun NH_2_-terminal kinase (JNK), p53 up-regulated mediator of apoptosis (PUMA)

## Abstract

**Background:**

*Artemisia capillaris* (AC) has been recognized as one of the promising candidates for hepatoprotective, hypoglycemic, hypolipidemic, antiobesitic and anti-inflammatory therapeutic effectiveness. This study evaluated the inherent mechanism and anti-apoptotic activity of 30% ethanol extract of AC (AC extract) 100 μg/ml on free fatty acids (FFAs)-induced HepG2 cellular steatosis and lipoapoptosis.

**Methods:**

Hepatic steatosis was induced by culturing HepG2 cells with a FFAs mixture (oleic and palmitic acid at the proportion of 2:1) for 24 h, thus ultimately giving rise to lipoapoptosis. Cell viability and lipid accumulation were detected by MTT assay and Oil Red O staining method respectively and Caspase-3, −9, Bax, Bcl-2, p-JNK and PUMA were measured for lipoapoptosis after 24 hours.

**Results:**

AC extract significantly improved the FFAs-induced steatosis without cytotoxicity and Caspase-3, −9, Bax and Bcl-2 were modulated profitably to HepG2 cells after AC treatment. In addition, AC extract inhibited the activation of c-Jun NH_2_ terminal kinase (JNK) and PUMA, which mechanism is related to non-alcoholic steatohepatitis (NASH).

**Conclusions:**

Combined together, AC extract exerted an obvious hypolipidemic and anti-apoptotic effect, indicating that AC extract might have potential therapeutic herb against NASH.

## Background

The accumulation of fat in the liver is pathogenic if total lipid exceeds more than 5% of liver weight or hepatocytes containing intracellular fat droplets are above 5% [1]. Alcohol is one of well-known causes for this higher intrahepatic fat content called fatty liver. In recent times, however, non-alcoholic fatty liver disease (NAFLD) without excessive drinking of alcohol (<20 g/d for female and 30 g/d for male) draws public attention [2].

The prevalence of NAFLD is estimated to be about 34% among adults in the United States [3] and it is recognized as the primary cause of liver dysfunction in children [4]. Concerning this common liver disease, it encompasses a variety of hepatic features from simple fat deposition to non-alcoholic steatohepatitis (NASH), fibrosis, severe cirrhosis and hepatocellular carcinoma (HCC). About 10% of benign steatosis will develop into more severe NASH [5], which indicates the characteristic increase of inflammatory and apoptotic cells in the liver, and may result in cirrhosis up to 25% [6]. In addition, NASH was reported to be the third commonplace disease to which liver transplantation is efficacious [7] and patients with NASH exhibit substantially high mortality in cardiovascular diseases [8]. Nevertheless, the current medical world has no validated treatment for NASH [9].

There are various tools that may explain the pathogenesis and progress of NASH: endoplasmic reticulum (ER) stress, oxidative stress, inflammatory factors, insulin resistance, and so on. These days, however, increasing evidence suggests that ectopic fat incretion in liver triggers lipoapoptosis [10], a potential underlying mechanism involved in apoptosis in NASH [11], which is shown in NASH liver cell under free fatty acids (FFAs) overload [12]. Thus, lipoapoptosis is a distinguishable character in human with NASH in that its feature was more remarkable in NASH than simple steatosis [13] and alcoholic steatohepatitis [12]. For this programmed cell death by excess lipid deposition in NASH, a lot of reports have expressed c-Jun NH_2_terminal kinase (JNK) as a potential modulator activating apoptotic effectors such as p-53-up-regulated mediator of apoptosis (PUMA), Bax, Caspase-3 and −9. In other words, JNK activation by FFAs can induce mitochondrial apoptotic pathway by increasing PUMA expression which modulates the pro-and anti-apoptotic proteins such as Bax and Bcl-2 [14]. Judging from this lipoapoptotic pathway, PUMA and JNK could be specific targets for treatment of NASH.

*Artemisia Capillaris* (AC), which is included in the family of Asteraceae and belongs to the plant genus *Artemisia*, is an indigenous medicinal herb widely used as hepatoprotective, analgesic, and antipyretic drug [15]. As metabolic syndromes, such as dyslipidemia, hyperglycemia, obesity and cardiovascular disease have been major public health problem, relevant approaches to the therapeutic activities of AC are noteworthy. For example, AC has anti-diabetic and lipid-lowering effects in hyperglycemic patients [16] and dyslipidemic rodents [17] as well as HepG2 cell incubated with 1 mM palmitic acid (PA) [18] respectively and AC ethyl acetate fraction decreased the accumulation of body fat by suppressing PPAR γ in adipocytes [19]. Furthermore, AC contributed to anti-fibrotic [20], anti-oxidant [21] and anti-inflammatory [22] effects involved in the pathological feature of NASH.

Despite this therapeutic suggestion of AC for NASH, there have not been yet extensive studies that explain medical connection between AC and NASH in respect of JNK and PUMA. In this regard, this study was designed to examine the anti-steatotic and anti-apoptotic effects of 30% ethanol extract of AC (AC extract) on HepG2 cells induced by FFAs 1 mM to show the effectiveness for NASH. We measured the PUMA down-regulatory effect of AC extract and tried to investigate the inhibitory effect of AC extract on JNK signaling linked to PUMA, a key pathway relevant to lipoapoptosis. Furthermore, the result from this NASH model will contribute to developing a potential therapy for human NASH.

## Methods

### Preparation of AC extract

AC was purchased from Kyung Hee Oriental Herbal Medicine Research Center (Seoul, South Korea). The herb was cut down in a proper size, and extracted as follow. Above all, 30% EtOH was added to AC 100 g and then 2 times extracted repeatedly for 3 h at 40°C using extractor (JAC-4020, KODO Technical Research Co., Ltd., Hwaseong, South Korea). After vacuum evaporation (N-1000S-WD, Eyela Co., Tokyo, Japan) of this sample, it was dissolved with 30% EtOH 100 ml at 4°C for 24 h. And then, supernatant liquid of the solvent was freeze-dried (FDU-1100, Eyela Co., Tokyo, Japan) after one more vacuum evaporation, thereby yielding the 30% ethanol extract of AC (AC extract) [23].

### HepG2 cell culture

Fully differentiated human hepatoblastoma cell line, HepG2 cells were purchased from the Korean Cell Line Bank (KCLB®, Seoul, South Korea). HepG2 cells were grown for suspension culture at 37ºC in an atmosphere of 5% CO_2_ in a Dulbecco's Modified Eagle Medium (DMEM) containing 4.5 g/L of glucose (Lonza, Walkersville, MD) supplemented with 10% fetal bovine serum (FBS; Lonza, Walkersville, MD) and antibiotics antimycotics (Sigma-Aldrich, St. Louis, MO).

### Cell viability assay

MTT (3-(4,5-dimethythiazol-. 2-yl)-2,5-diphenyl tetrazolium bromide) was purchased from Invitrogen (Carlsbad, CA). Before treatment, it was dissolved as a 1 mg/ml stock in phosphate-buffered saline (PBS). HepG2 cells were seeded at a density of 1.5 × 10^3^ cells/well in 96-well plate and incubated for 48 h. The cells were treated with disparate concentrations of AC extract (100, 500 and 1000 μg/ml) for 24 h and FFAs 1 mM in another group for 1 h and 24 h, respectively. And then, 100 μl of MTT solution were treated for 2 h. After 4 h, MTT solution was removed by aspiration, the insoluble formazan crystals were dissolved in DMSO and measured the absorbance was read at 570 nm with a Bio-Rad model 680 microplate reader (Bio-Rad, Hercules, CA).

### FFAs and AC extract treatment

Oleic acid (OA) and PA were purchased from Sigma-Aldrich (St. Louis, MO). OA and PA were dissolved in isopropanol at the concentration of 50 mM stock solution. DMEM containing 1% bovine serum albumin (BSA; Lonza, Walkersville, MD) was used in this experiment. Final concentration of the both fatty acids was 50 μM. After starvation with DMEM containing low glucose for 24 h, FFAs 1 mM (OA 0.66 mM and PA 0.33 mM) were treated for 24 h. After treatment of FFAs, AC was treated at the concentration of 100 μg/ml for 24 h.

### Oil Red O staining

Oil Red O solution was purchased from Sigma-aldrich. Oil Red O staining was performed according to the reference [24,25]. To stain adipocytes, cells were washed three times with PBS to remove unbound staining and fixed with 10% formalin for 1 h. After washing for three times with distilled water, cells were washed with 60% isopropanol briefly and incubated with 60% filtered Oil Red O solution (0.7 g per 200 ml of isopropanol) for 30 min at room temperature. Cells were washed with water briefly and then stained with hematoxylin (Sigma-Aldrich, USA). For quantitative analysis of Oil Red O contents levels, isopropanol was added to each samples and then shaken at room temperature for 5 min. The absorbance was read at 510 nm with a Bio-Rad model 680 microplate reader.

### Western blot analysis

The cells were washed and scraped with PBS, and incubated in an RIPA buffer containing a protease inhibitor cocktail (Roche Diagnostics, Mannheim, Germany). After protein preparation, Bradford assay was performed. The same amounts of total protein (20 μg) were resolved in 12% sodium dodecyl sulfate (SDS)-acrylamide gel and transferred to the PVDF membrane. The following primary antibodies were used: PUMA, JNK, p- JNK, Caspase-3, −9, Bax and Bcl-2 1:3000 in 2% BSA (Cell signaling, Danvers, MA). β-actin (Santa Cruz Biotechnology, Inc., Dallas, TX) was used as the internal control. Membrane was incubated with the secondary antibody (1:10,000 dilution); the blot was detected with an ECL solution (EMD Millipore Corporation, Billerica, MA) using a Davinch-Chemi™ chemiluminescence imaging system (Davinch-K, Seoul, South Korea).

### Real-time quantitative PCR analysis

Total RNA was isolated from HepG2 cells using a Hybrid-R kit (GeneAll, Seoul, South Korea). Following that, the cDNA was hybridized from 1 μg of the total RNA with a LeGene 1st strand cDNA synthesis system (LeGene Bioscience, San Diego, CA). *BBC3* (PUMA) mRNA expression level was determined by a quantitative PCR as described in the manufacturer’s protocol (Life Technologies, Grand Island, NY). To analyze the results, 2^-∆∆Ct^ values compared to the normal sample were determined with StepOne software (Life Technologies, Grand Island, NY). *GAPDH* was used as an endogenous control. The sequences of the forward and reverse primer were 5′-CATGGCCTTCCGTGTTCCTA-3′ and 5′-GCGGCACGTCAGATCCA-3′ for the *GAPDH* gene, 5′- GACGACCTCAAC GCACAGTA-3′ and 5′- AGGAGTCCCATGATGAGATTGT-3′ for the PUMA gene, respectively [26,27].

### Statistical analysis

All data represent at least two separate experiments and each experiment was performed in triplicate. The significance of the data was analyzed with Prism 5 software with one-way ANOVA and Bonferroni’s post-hoc test to compare each set of data. Bars show the means ± SEMs. *, *p* < 0.05; **, *p* < 0.01; ***, *p* < 0.001.

## Results

### Effects of FFAs and AC extract on cell viability

**Figure 1:**
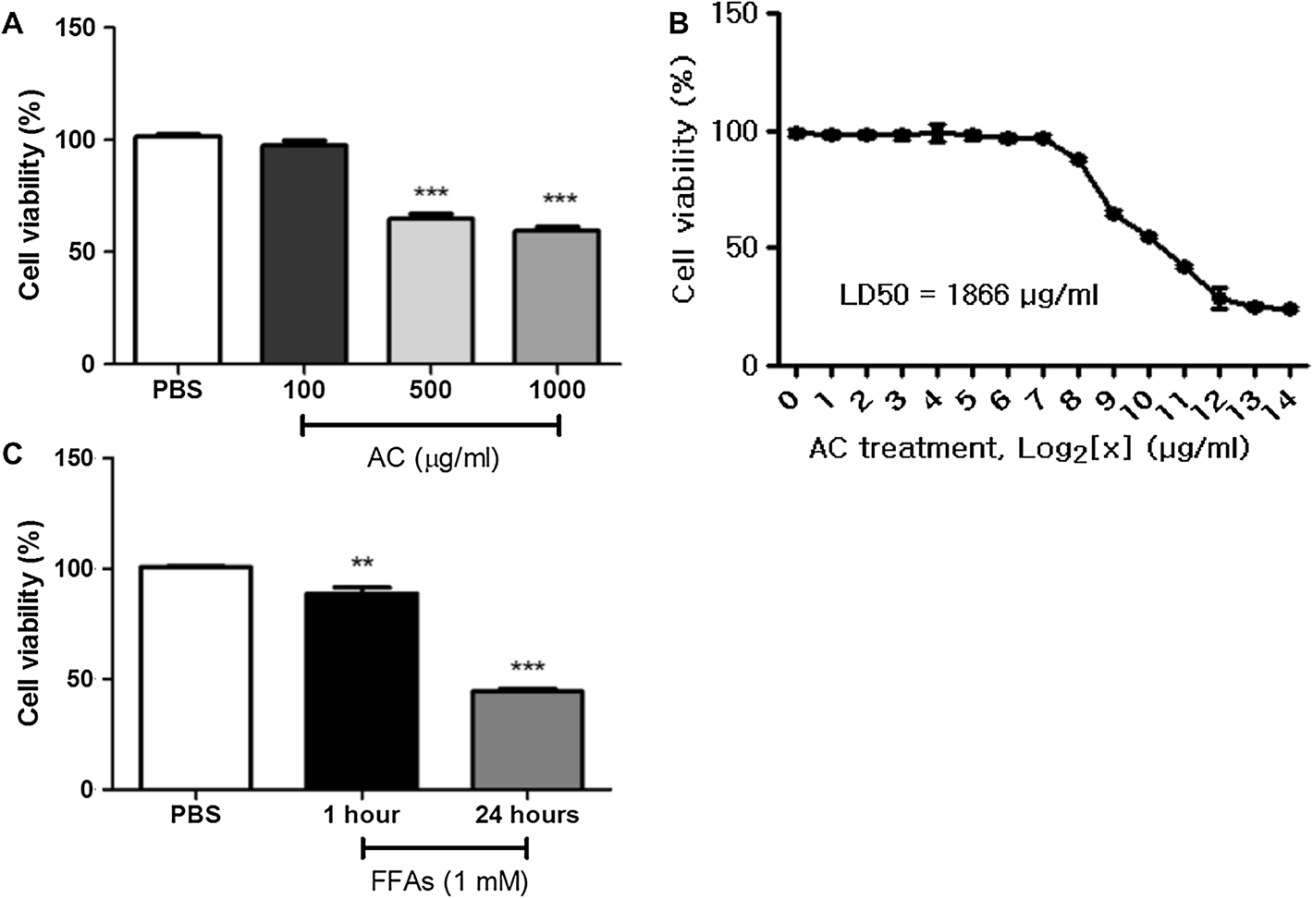
**Cell viability assay.** After treatment of AC extract and FFAs 1 mM on the HepG2 cells, MTT assay was performed. AC extract was treated as 100, 500 and 1000 μg/ml for 24 h. AC 100 μg/ml showed no toxicity to HepG2 cells **(A)**. Differently concentrated AC extracts were treated on HepG2 cells for 24 h and the LD50 was calculated **(B)**. FFAs were treated at 1 mM concentration for 1 h or 24 h. The treatment of FFAs showed significant toxicity to HepG2 cells for both 1 h or 24 h **(C)**. Statistical significance was determined by one-way ANOVA and the values are mean ± SEM. **, *p* < 0.01; ***, *p* < 0.001.

To determine whether the treatment of AC extract on HepG2 cells has value for medical use with no toxic effect, the cells were treated with different concentrations of AC extract (100, 500 and 1000 μg/ml) for 24 h and cell viability was evaluated by MTT assay. Both 500 and 1000 μg/ml of AC extract were significantly toxic to HepG2 cells which viability was reduced up to about 60% (*p* < 0.001). In contrast, 100 μg/ml of AC extract showed no substantial decrease in cell viability (Figure 1A). And we determined the LD50 of the AC extract on HepG2 cells. AC extract was treated with 15-different concentrations on HepG2 cells for 24 h, and the LD50 was calculated. The LD50 was 1866 μg/ml (Figure 1B).

As compared to AC extract, after treating HepG2 cells with FFAs 1 mM for 24 h, this lipid overload induced incomparably detrimental effect on cell viability. The number of viable cells significantly decreased to less than 50% in a time-dependent manner (*p* < 0.001) (Figure 1C). These data indicate that HepG2 cells undergo significant lipotoxic change under FFAs 1 mM for 24 h with about 50% decreased amount of viable cells as compared to the safety of AC extract treatment and the cells overexposed to FFAs could be *in vitro* model of hepatic lipoapoptosis.

### Effect of AC extract on steatosis

**Figure 2:**
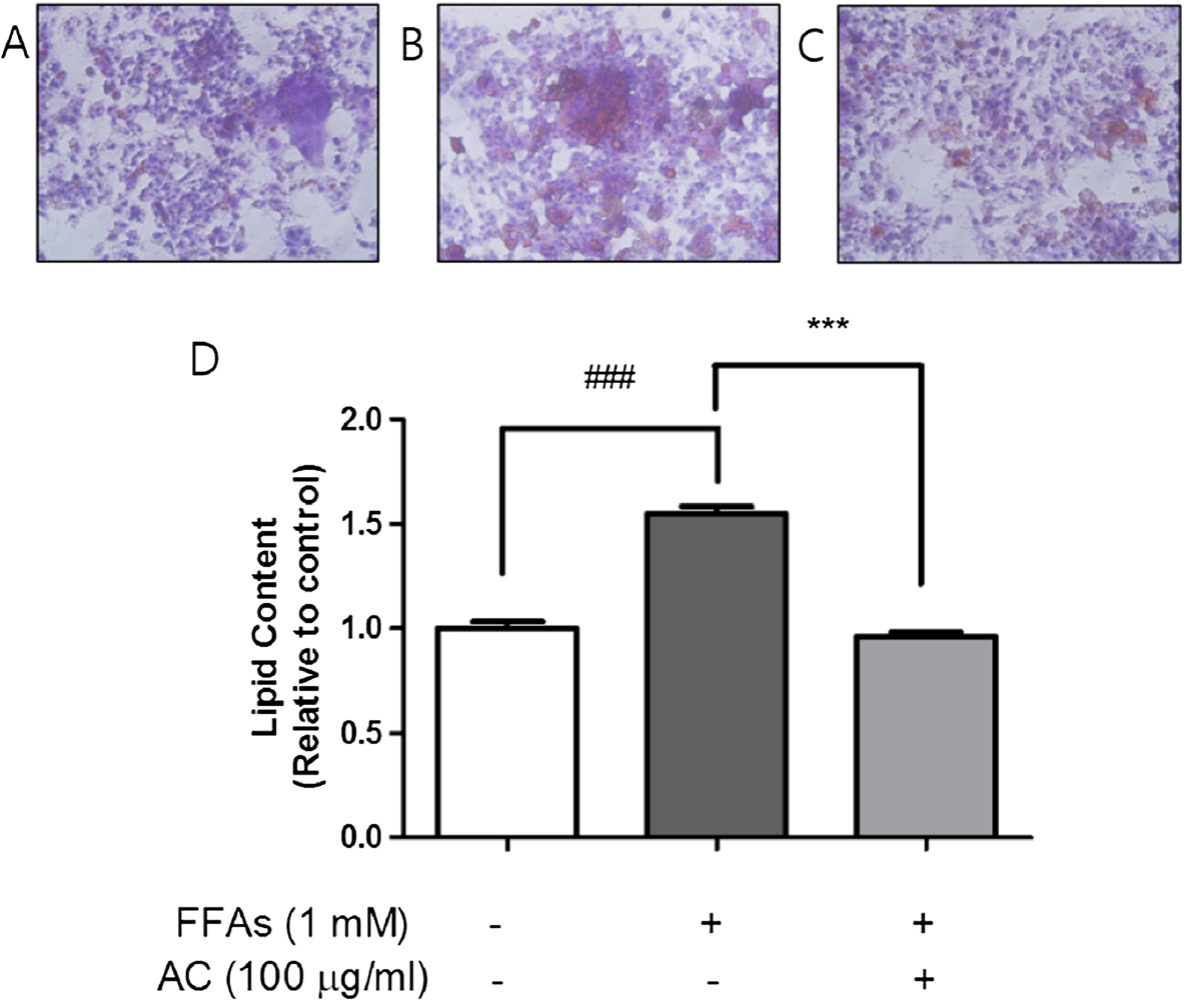
**Oil Red O staining for HepG2 cells and quantitative analysis of lipid.** FFAs 1 mM for 24 h induced lipid accumulation in HepG2 cells. Visual observation of lipid content was captured by microscope (×400): the control cells treated with only 1% BSA **(A)**, cells treated with FFAs 1 mM for 24 h **(B)**, and cells pretreated with FFAs 1 mM for 24 h and then cultured with AC extract for 24 h **(C)**. The quantitative analysis of cellular steatosis **(D)** was measured through deposited Oil Red O in the cells. Statistical significance was determined by one-way ANOVA and the values are mean ± SEM. ###, *p* < 0.001, control versus FFAs-treated group (lipoapoptosis-induced group) and ***, *p* < 0.001, FFAs-treated group (lipoapoptosis-induced group) versus AC extract treated group.

In order to observe hepatic lipid accumulation, HepG2 cells were exposed to FFAs 1 mM, the mixture with two fatty acids which co-incubation can lead to steatogenesis and apoptosis simultaneously in hepatocytes. In other words, this FFAs organization with OA and PA 2:1 enables fat contents to be maximized and minimizes cellular damage induced by lipid overload tolerating some degree of apoptosis [25]. After culturing with FFAs 1 mM for 24 h in media containing 1% BSA, HepG2 cells were stained with Oil Red O solution for 30 min at room temperature, and then the increased intracellular lipid contents dyed pink were visually observed by microscope (×400) (Figure 2B). This lipid deposition stained by Oil Red O was analyzed quantitatively and a bar graph to display the results confirmed the visible lipid incretion versus only 1% BSA to be statistically significant (*p* < 0.001). In this FFAs-induced steatosis in HepG2 cells, the high buildup of lipid droplets was ameliorated to their almost original condition after treatment of AC extract for 24 h (Figure 2C and D).

Steatosis is one of hallmark properties of patients with NASH [28]. Accordingly, the significantly reduced cellular lipid level in AC extract-treated group could show the potential for the development of appropriate treatments for NASH.

### Effect of AC extract on pJNK activation

**Figure 3:**
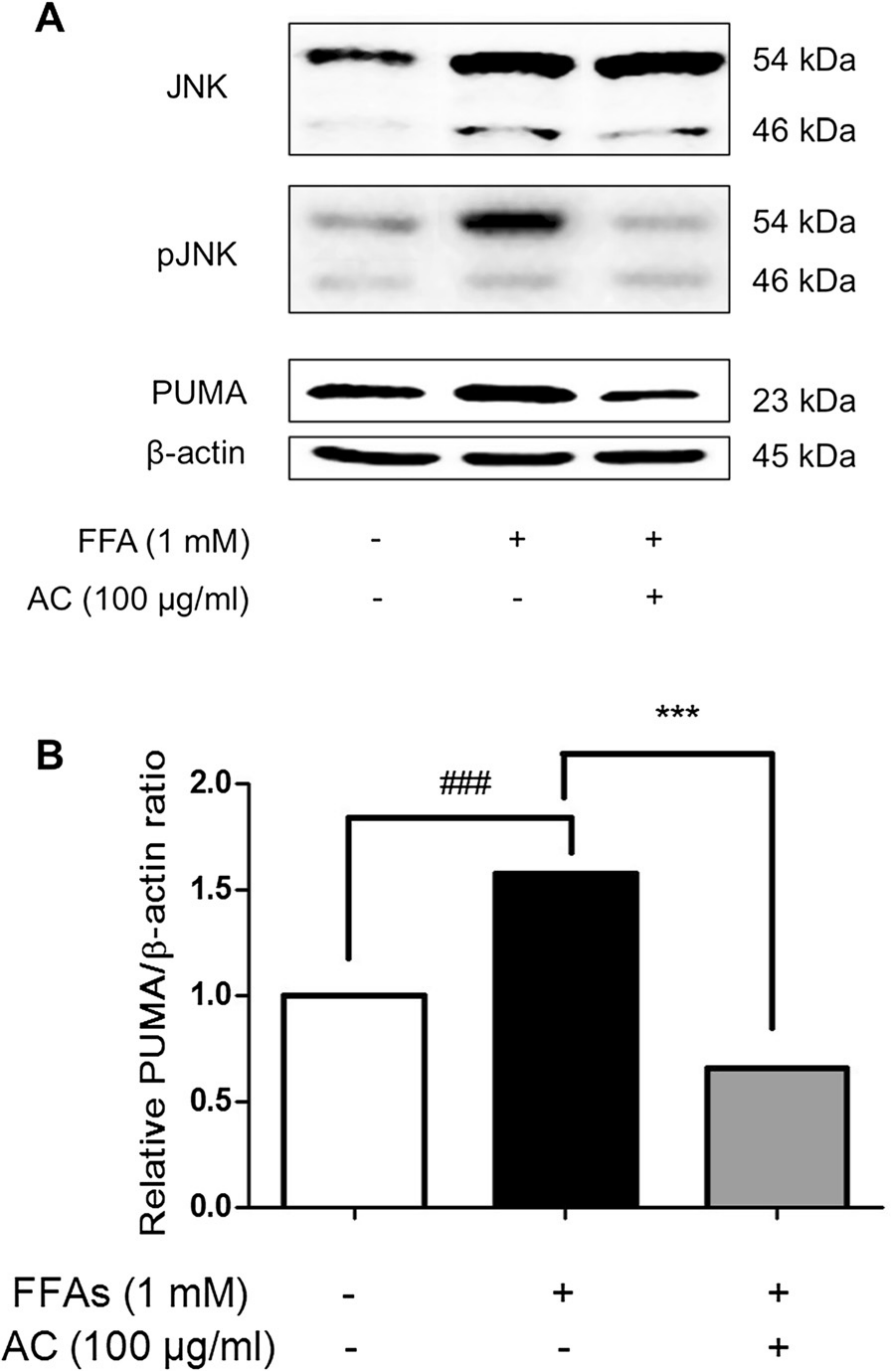
**pJNK and PUMA expression level.** Total cell lysates were prepared from control cells, cells treated with FFAs 1 mM for 24 h, and cells pretreated with FFAs 1 mM for 24 h and then cultured with AC extract for 24 h respectively. **(A)** The overexpressed level of pJNK and PUMA were down-regulated after treatment of AC extract for 24 h. **(B)** PUMA expression level normalized with β-actin was measured by densitometry analysis and represented as bar charts. β-actin was used as an internal control. Statistical significance was determined by one-way ANOVA and the values are mean ± SEM. ###, *p* < 0.001, control versus FFAs-treated group (lipoapoptosis-induced group) and ***, *p* < 0.001, FFAs-treated group versus AC extract treated group.

Since the increase in pJNK expression is a principal characteristic in HepG2 cells under lipid overburden [14] and JNK is triggered in the liver tissue of NASH patients [27], the regulation of pJNK might open the therapeutic way for NASH, a chronic liver disease. Based on western blot using primary antibody for pJNK, treatment on HepG2 cells with total FFAs 1 mM for 24 h evidently amplified the pJNK expression level (Figure 3A). This result is consistent with the previous studies in which JNK activation contributes to the lipoapoptosis observed in lipogenic hepatocyte damage [14]. Compared with the FFAs-treated group, AC extract exposure following pretreatment with FFAs 1 mM generated a conspicuous decrease in pJNK activity, which had been augmented by FFAs for 24 h (Figure 3A). As a consequence of this, the post-treated AC induced inhibitory effect on JNK phosphorylation which hampers lipid metabolism and develops into NASH. In other words, the presence of AC extract disturbed JNK-mediated pathway in hepatocyte during lipoapoptosis and JNK-dependent cascade can be a hypothesized pathway to explain the mechanism through which AC extract contributes to the improvement of FFA-mediated lipoapoptosis in HepG2 cells.

### Effect of AC extract on PUMA activation

**Figure 4:**
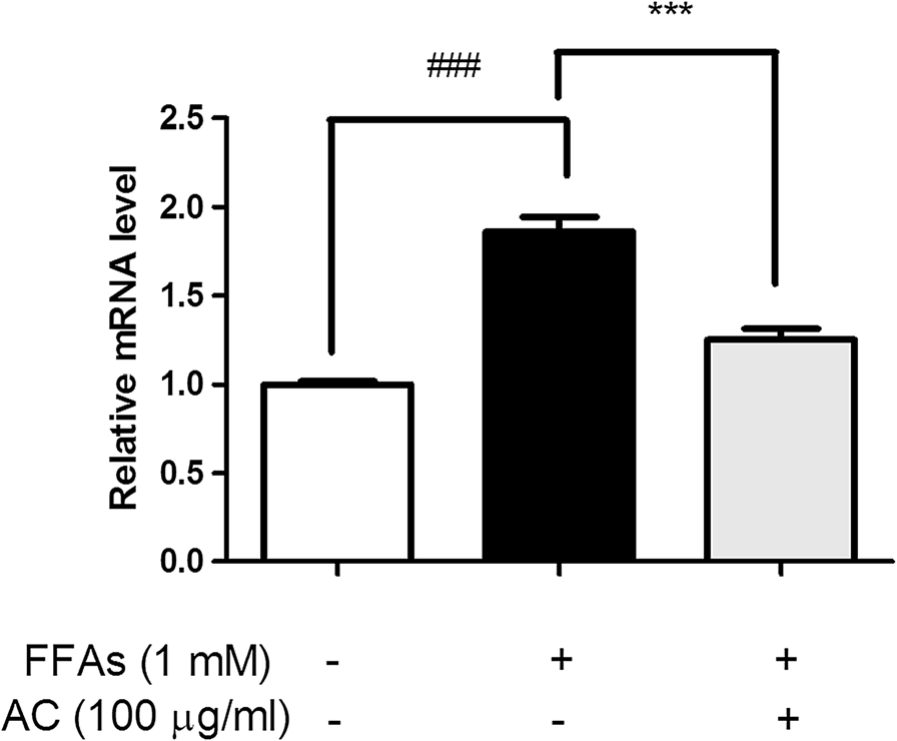
**PUMA mRNA expression level.** PUMA mRNA was prepared from control cells, cells treated with FFAs 1 mM for 24 h, and cells pretreated with FFAs 1 mM for 24 h and then cultured with AC extract for 24 h, respectively. Data were normalized with *GADPH* level. Statistical significance was determined by one-way ANOVA and the values are mean ± SEM. ###, *p* < 0.001, control versus FFAs-treated group (lipoapoptosis-induced group) and ***, *p* < 0.001, FFAs-treated group versus AC extract treated group.

FFAs could cause cellular injury through JNK-potentiated route which mechanism is complicated. The possible leading pathway of this lipoapoptotic procedure is intimately correlated with a series of sequence from pJNK to proapoptotic proteins. PUMA, a pro-apoptotic protein, is up-regulated via JNK phosphorylation and then stimulates Bax [27], which leads to mitochondrial apoptotic response depending on caspase activity [29]. In particular, PUMA expression is highly related to FFAs-overloaded lipoapoptosis in hepatic cells [27]. Actually, PUMA mRNA and protein levels increased significantly over the control group at 24 h after FFAs 1 mM treatment in HepG2 cells (Figures 3 and 4). Interestingly, the 100 μg/ml of AC extract at which dosage hepatic steatosis had been effectively improved (Figure 2), also attenuated the increased PUMA as well as pJNK (Figures 3 and 4). These data suggest that AC extract might regulate PUMA transcription in hepatic lipoapoptosis and play an inhibitory role in an apoptotic axis associating with JNK and PUMA.

### Effect of AC on Bax, Bcl-2 and Caspase activation

**Figure 5:**
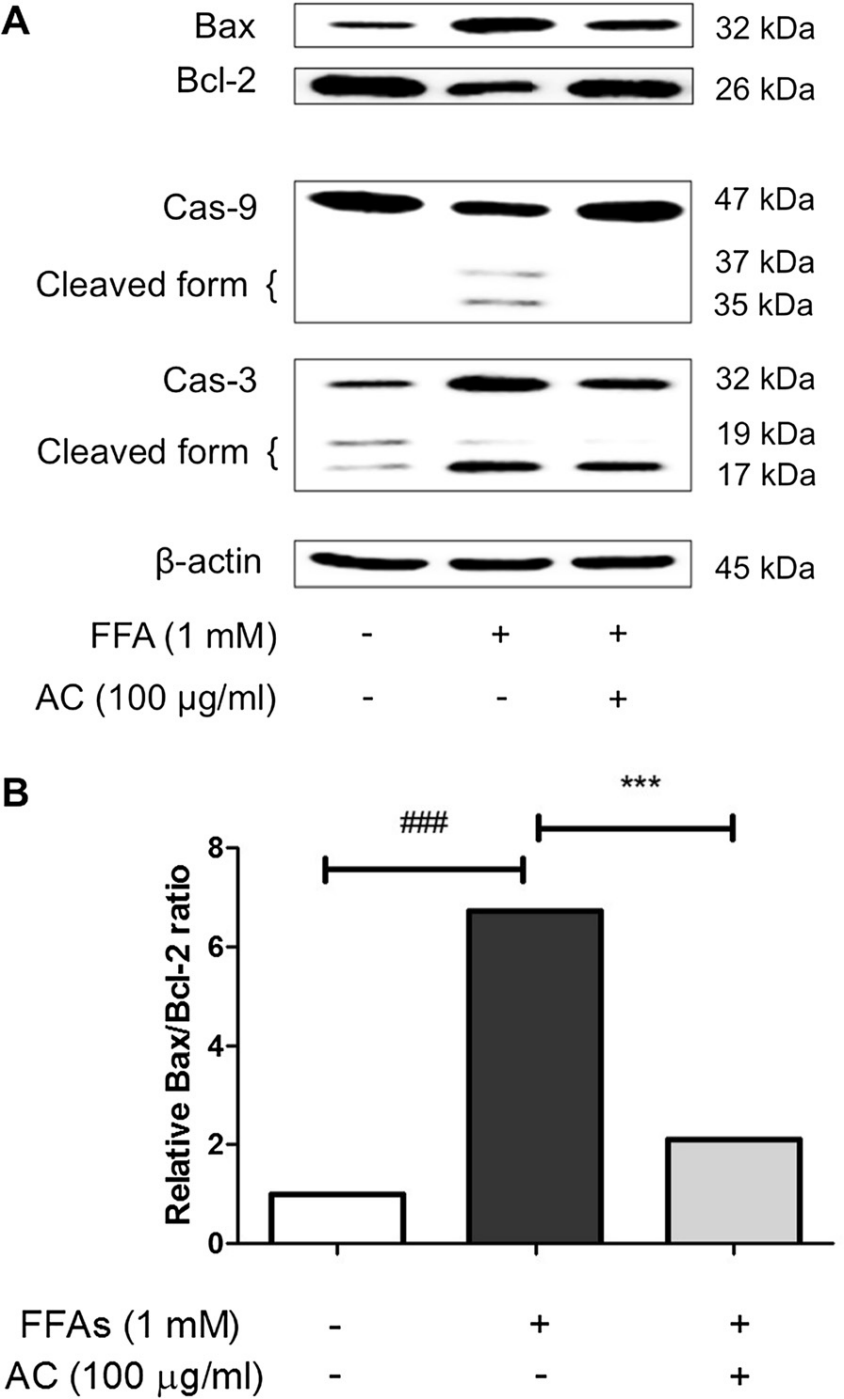
**Bax, Bcl-2, Caspase-9, and −3 expression level.** Bax and Caspase-3 were activated while Bcl-2 and Caspase-9 were suppressed conspicuously after FFAs 1 mM treatment in HepG2 cells. But, AC extract treatment for 24 h reduced the expression level of Bax and Caspase-3, and augmented the Bcl-2 and Caspase-9 manifestation **(A)**. In a bar graph, AC treatment suppressed the Bax/Bcl-2 expression level which had been significantly increased after induction of lipoapoptosis **(B)**. The cleaved forms of Caspase-3 and Caspase-9 were down-regulated after treatment of AC extract **(A)**. Statistical significance was determined by one-way ANOVA and the values are mean ± SEM. ###, *p* < 0.001, control versus FFAs-treated group (lipoapoptosis-induced group) and ***, *p* < 0.001, FFAs-treated group versus AC extract treated group.

The activation of PUMA proteins under lipotoxic situation was known to suppress Bcl-2, an anti-apoptotic protein, and function to release cytochrome c from the mitochondria into the cytosol, thereby activating Caspase-3 and −9 [30] and PA-induced PUMA expression enhanced Bax activation in lipoapoptosis [27]. In addition, Bax activation and lipoapoptosis depend on JNK downstream [31]. To confirm the effect of AC extract on HepG2 cells depending on this sequential process in lipoapoptosis, Bax, Bcl-2, Caspase-3 and −9 were finally analyzed by western blotting method in this experimental model. After HepG2 cells were overexposed to FFAs 1 mM for 24 h, Bax, Caspase-3 and −9 activations were observed as anticipated (Figure 5A). In particular, Bax/Bcl-2 ratio reached an about 7-fold increment as compared to the control group (Figure 5B). Following AC extract treatment for 24 h after inducing lipoapoptosis in HepG2 cells, western blot analysis showed that AC extract significantly blocked considerable cellular death by inhibiting activation of Bax and catalytic cleavage of Caspase-3 and −9 (Figure 5A). Taken together, lipoapoptosis induced by FFAs 1 mM in HepG2 cells undergoes the mitochondrial pathway involved in the activation of apoptotic executors as Bax, Caspase-3 and −9 and AC treatment might be revealed to be efficient in restoring hepatic injury.

## Discussion

The adipocyte has the effective function which disposes large amounts of FFAs. However, most other cells experience the harmful impairment, referred to as lipoapoptosis, under the FFAs overload. In other words, lipid surplus in non-adipose tissue produces detrimental change, thereby eventually leading to cellular damage and death [32]. In case of hepatocytes, fat-laden liver cells overloaded with FFAs may bring about severe fatty hepatitis, a metabolic syndrome called NASH. A pathogenesis of this NASH is as follows. Originally, as the moderate amount of circulating FFAs enters liver cells, these FFAs were changed to detoxified neutral triglycerides [27]. However, the patients with NASH show more elevated circulating FFAs than NAFLD people [33]. This high non-esterified circulating FFAs concentration in NASH may aggravate the toxicity and lipoapoptosis to liver.

To make the closest *in vitro* model to hepatocellular lipoapoptosis in NASH, HepG2 cells and FFAs 1 mM were applied in this present study. Although human hepatic cells like L-02 (Chu, Wang et al. 2011), LX-1 and LX-2 [34] seem to be strictly representative of human liver, there are some barriers such as difficulty in reproducing [35]. On the other hand, HepG2 or Huh-7 cells are easily available with numerous replications but can lead to instable genetic and epigenetic modification. [36]. In particular, HepG2 cells, a well differentiated human hepatoblastoma cell line, are widely used because they can express various functions related to liver [37]. In addition, both FFAs-overloaded human hepatocytes and HepG2 cells exhibited comparable levels of intracellular lipid contents, which were nearly similar to lipid accumulation of the hepatocytes obtained from human steatotic liver [38]. Accordingly, HepG2 cells could be reliable alternative cell lines to make the realistic NASH experimental model.

OA and PA are representative of FFAs in liver of both normal subjects and NAFLD patients [39]. Steatosis extent was larger when cells were treated with OA than PA and the cellular susceptibility and toxicity to lipid is more severe in PA than OA [25]. In this context, an appropriate mixing ratio of PA and OA can lead to significant lipid intracellular accumulation and lipoapoptosis, but just minimizing cellular damage. In one recent study, FFAs 1 mM consisted of OA and PA 2:1 maximized fat accumulation without severe cellular toxicity [38]. Given this information, OA 0.66 mM and PA 0.33 mM were used in the present study because this combination proportion seems to be more efficient to induce lipoapoptosis in HepG2 cells than when used individually.

In this *in vitr*o model of lipoapoptosis, we investigated whether AC extract alleviates FFAs-induced lipoapoptosis in HepG2 cells. Since *in vitr*o model comfortably enables us to know the molecular pathway and curative power of drugs even though the remodeling of delicate human organ is difficult because of its rather simplicity [35], the current study is helpful for us to understand anti-steatic and anti-apoptotic effects of AC extract in respect of molecular mechanism.

It is preferential to elicit how a molecular pathway modulates the pathogenesis and progression of NASH for therapeutic use of AC extract. There are a large number of molecular algorithms which have been hypothesized to explain the lipid-mediated apoptosis: the extrinsic pathway associated with Fas ligand or tumor necrosis factor receptor and the mitochondrial-mediated intrinsic pathway [25]. According to the current studies, activation of JNK, a stress-stimulated member of mitogen-activated protein kinase (MAPK) family, has been regarded as the most probable pro-apoptotic mechanism in lipid-mediated apoptosis. For example, lysophosphatidylcholine treatment resulted in a robust activation of JNK [40] and JNK modulated Bax activation under cellular toxicity induced by saturated fatty acids [14]. In addition, JNK is well expressed in the liver stressed by lipid. JNK is activated in human NASH as well as murine models of steatohepatitis [41,42]. Moreover, JNK inhibitors abrogate lipoapoptosis in both human and mouse hepatic cell lines [14]. Therefore, lipoapoptosis in liver cell line has been linked to induction of JNK and pJNK is highly expressed after FFAs 1 mM treatment on HepG2 cells. Interestingly, stimulation of JNK influences on the subsequent increase in PUMA and both JNK and PUMA expression are closely related to lipid overload of the NASH people [27]. PUMA, p53 inducible gene, is closely related to strong apoptosis and modulates anti-apoptotic Bcl-2 and pro-apoptotic Bax, thereby activating caspase-3 and −9 [30]. In addition, it has been reported that PUMA over-activation is associated with p53-dependent lipoapoptosis in mitochondria [43]. Furthermore, the progress of apoptosis continues at a rapid pace within hours after PUMA expression [44].

In this connection, we focused our study on the relation between AC extract treatment and PUMA and it may be summarized as follows. AC extract treatment (100 μg/ml) significantly lowered PUMA mRNA and protein levels as well as pJNK (Figures 3 and 4). This is a novel finding that shows the junction of PUMA and AC extract in NASH *in vitro* model, powerfully suggesting the therapeutic potential of AC extract on NASH. There are two major lipid-lowering and anti-apoptotic action in AC extract on excess FFAs in HepG2 cells. First, treatment of AC extract considerably decreased accumulation of lipid droplets in HepG2 cells, not affecting their viability. Although buildup of lipid to some degree, especially in case of oleic acids, protects the cells from lipotoxicity [25], the lipid-reducing operation of AC extract in this study might be explained as cytoprotective and antiapoptotic effects because this FFAs surplus is accompanied by apparent apoptosis, which executors like Bax and Caspase were highly identified by Western blotting analysis (Figure 5). Secondly, we confirmed that AC extract treatment recovered the FFAs-induced Bax, caspase, PUMA and pJNK, suggesting that AC extract influences on mitochondrial apoptotic pathway (Figure 3). There is little information on medicinal drugs which have down-regulatory effect on PUMA in relation to NASH. Additionally, the function of AC extract on PUMA and pJNK in lipid-induced liver cells has not been studied yet.

## Conclusions

In conclusion, AC extract (100 μg/ml) alleviated hepatic steatosis induced by the accumulation of FFAs 1 mM in HepG2 cells, indicating that it promoted the ability of disposing lipid and blocked hepatic lipid pile, and decreased pJNK, PUMA, Bax and caspase relevant to apoptosis. Based on this intriguing finding, PUMA and pJNK might give molecular hint on developing AC extract as validated regimen against NASH. Even though there are some limitations like the use of HepG2 cells, not human liver cells, and no data on the pre-treatment of AC extract for cytoprotective effects and comparison with Vitamin E [45] or antidiabetic metformin [46] possible as a potential treatment for NASH, our study would give an applicable idea useful to therapeutic strategies of AC extract in NASH. This current study suggests that AC extract might be a candidate treatment for further research in *in vivo* NASH model, thereby providing some critical information that enables to understand the progression of steatohepatitis toward cirrhosis and liver cancer.

## Abbreviations

AC: *Artemisia capillaris*; FFAs: Free fatty acids; JNK: c-Jun NH_2_-terminal kinase; NASH: Non-alcoholic steatohepatitis; PUMA: p53 up-regulated mediator of apoptosis; NAFLD: Non-alcoholic fatty liver disease; ER: Endoplasmic reticulum; OA: Oleic acid; PA: Palmitic acid.

## Competing interests

The authors have declared that there are no competing interests.

## Authors’ contributions

E-GJ, M-HS, K-SK carried out the *in vivo, in vitro* experiments and drafted the manuscript. H-JW, Y-CK, J-HL designed the study and performed the statistical analysis. Y-CN, Y-MK carried out chemical analysis of AC. H-JJ performed the statistical analysis and drafted the manuscript. All authors read and approved the final manuscript.

## Pre-publication history

The pre-publication history for this paper can be accessed here:

http://www.biomedcentral.com/1472-6882/14/253/prepub
